# Primary mediastinal large B‐cell lymphoma

**DOI:** 10.1002/jha2.151

**Published:** 2020-12-10

**Authors:** Diego Villa, Laurie H. Sehn

**Affiliations:** ^1^ Centre for Lymphoid Cancer and Division of Medical Oncology BC Cancer and The University of British Columbia Vancouver British Columbia Canada

A 27‐year‐old woman presented with dyspnea and chest pain. Computed tomography scan showed an 18 cm anterior mediastinal mass compressing the heart and left lung, with tracheal deviation and chest wall extension. There was complete left lung atelectasis and a pleural effusion occupying the entire hemithorax (Panel A). Positron‐emission tomography (PET) scan showed the mass contiguous with a circumferentially‐thickened left pleura, with images highlighting the PET‐avidity of this disease (Panels B and C). Biopsy of the mediastinal mass revealed a diffuse infiltrate of CD20‐positive large B‐cells confirming a diagnosis of primary mediastinal large B‐cell lymphoma (PMBCL). She was treated with six cycles of dose‐adjusted etoposide, prednisone, vincristine, cyclophosphamide, doxorubicin, and rituximab (DA‐EPOCH‐R). End‐of‐treatment PET showed a complete metabolic response (Panel D). PMBCL is an aggressive non‐Hodgkin lymphoma typically presenting in adults aged 20‐50, with female predominance. Urgent immunochemotherapy is indicated in patients with life‐threatening compression of thoracic structures and is potentially curative.


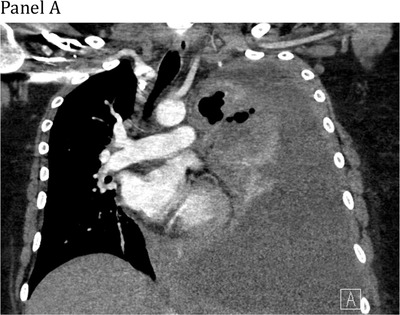



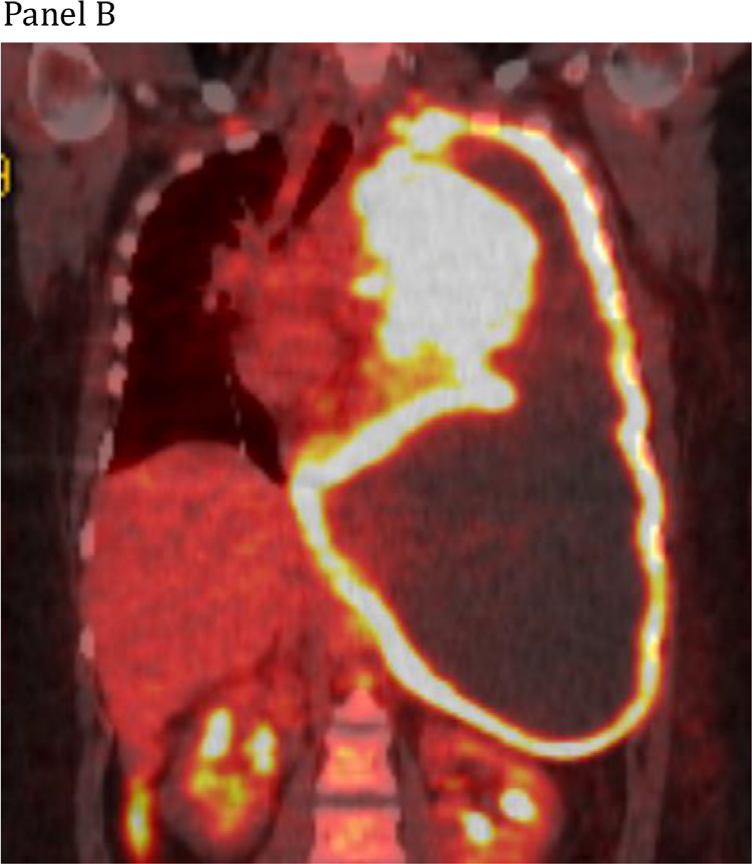



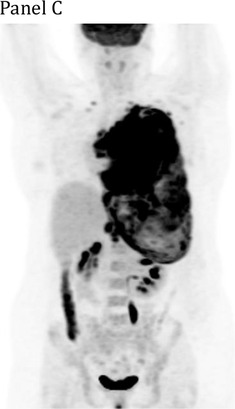



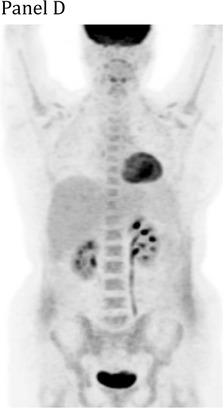


## AUTHOR CONTRIBUTIONS

Diego Villa and Laurie H. Sehn wrote the paper.

## PATIENT CONSENT STATEMENT

Authors have obtained written and verbal informed consent from the patient.

